# Nerve injury drives a heightened state of vigilance and neuropathic sensitization in *Drosophila*

**DOI:** 10.1126/sciadv.aaw4099

**Published:** 2019-07-10

**Authors:** Thang M. Khuong, Qiao-Ping Wang, John Manion, Lisa J. Oyston, Man-Tat Lau, Harry Towler, Yong Qi Lin, G. Gregory Neely

**Affiliations:** 1 The Dr. John and Anne Chong Laboratory for Functional Genomics, Charles Perkins Centre and School of Life and Environmental Sciences, The University of Sydney, NSW 2006, Australia.; 2 School of Pharmaceutical Sciences (Shenzhen), Sun Yat-sen University, Guangzhou 510275, China.; 3 Genome Editing Initiative, The University of Sydney, NSW 2006, Australia.

## Abstract

Injury can lead to devastating and often untreatable chronic pain. While acute pain perception (nociception) evolved more than 500 million years ago, virtually nothing is known about the molecular origin of chronic pain. Here we provide the first evidence that nerve injury leads to chronic neuropathic sensitization in insects. Mechanistically, peripheral nerve injury triggers a loss of central inhibition that drives escape circuit plasticity and neuropathic allodynia. At the molecular level, excitotoxic signaling within GABAergic (γ-aminobutyric acid) neurons required the acetylcholine receptor *nAChRα1* and led to caspase-dependent death of GABAergic neurons. Conversely, disruption of GABA signaling was sufficient to trigger allodynia without injury. Last, we identified the conserved transcription factor twist as a critical downstream regulator driving GABAergic cell death and neuropathic allodynia. Together, we define how injury leads to allodynia in insects, and describe a primordial precursor to neuropathic pain may have been advantageous, protecting animals after serious injury.

## Teaser

Nerve injury leads to loss of central inhibition and neuropathic pain in the fruit fly.

## INTRODUCTION

Chronic pain has an enormous impact on the quality of life for billions of patients, families, and caregivers worldwide, and current therapies do not adequately address pain for most patients (*2*). Globally, chronic pain is estimated to cost trillions of dollars per year (*3*), similar to the cost of cancer, heart disease, or diabetes. Lack of effective treatments for chronic pain has had knock-on effects in our society, for example, the opioid epidemic, where since 2000, >200,000 people have died from prescription opioid overdoses. Neuropathic pain (e.g., sciatica, back pain, cancer pain, diabetic pain, and accidental injury) is generally refractory to available therapies, with first-line antineuropathics providing adequate pain relief for only ~25% of patients (*4*). Despite decades of research into the molecular and physiological mechanisms that contribute to neuropathic pain, it is still not completely clear what we should target to treat the underlying pathology responsible for neuropathic pain. A basic understanding of the conserved architecture driving neuropathic pain may help us develop better, nonaddictive pain therapies that can reverse, or even resolve, chronic disease (*5*).

Nociception is the sense that allows animals to detect and escape potentially damaging stimuli that could adversely affect survival. In higher organisms, nociceptive sensory information is integrated and processed in the central nervous system (CNS) where pain is then experienced. Nociception first evolved more than ~500 million years ago (*1*), and the genetic architecture of this process appears to be under strong selective pressure (*6*–*10*). For example, transient receptor potential (TRP) channels, now central to our understanding of mammalian pain, were first described in the fruit fly *Drosophila melanogaster* (*11*, *12*), and this system has been a powerful tool for defining the core conserved genetic architecture of acute nociception from flies to humans (*6*, *7*, *10*, *13*–*15*). While much work has been done characterizing acute or transient nociceptive sensitization in the fly larvae (*16*, *17*), investigating chronic pain–like states has not yet been possible.

In humans, nerve injury can lead to neuropathic pain (*18*), where patients experience pain from innocuous sensory input (neuropathic allodynia); however, neuropathic sensitization is not strictly a vertebrate phenomenon. For example, injury can result in nociceptive sensitization in the marine mollusk *Aplysia* (*19*–*22*) or the squid *Doryteuthis pealei* (*23*), and injured animals acquire enhanced escape responses to natural predators, suggesting that neuropathic sensitization may confer a survival advantage (*24*). Much of what is known about invertebrate neuropathic sensitization has focused on neurophysiology, and the molecular mechanisms involved in neuropathic sensitization are completely unknown.

Here, we report that injury can lead to neuropathic allodynia in insects. We found that intact animals displayed a robust escape response to temperatures above 42°C; however, after injury, neuropathic thermal allodynia was observed, with escape responses showing a long-lasting shift to subnoxious temperature. To understand functional changes within the adult nociception circuit, we developed a nociception escape circuit electrophysiology preparation and, through this, observed enhancement of the escape circuitry after neuropathic injury. By combining these novel behavioral and electrophysiological assays with the *Drosophila* genetic toolbox, we provide the first genetic dissection of neuropathic sensitization in an invertebrate.

## RESULTS

### Neuropathic sensitization is a conserved response to injury

**Fig. 1:**
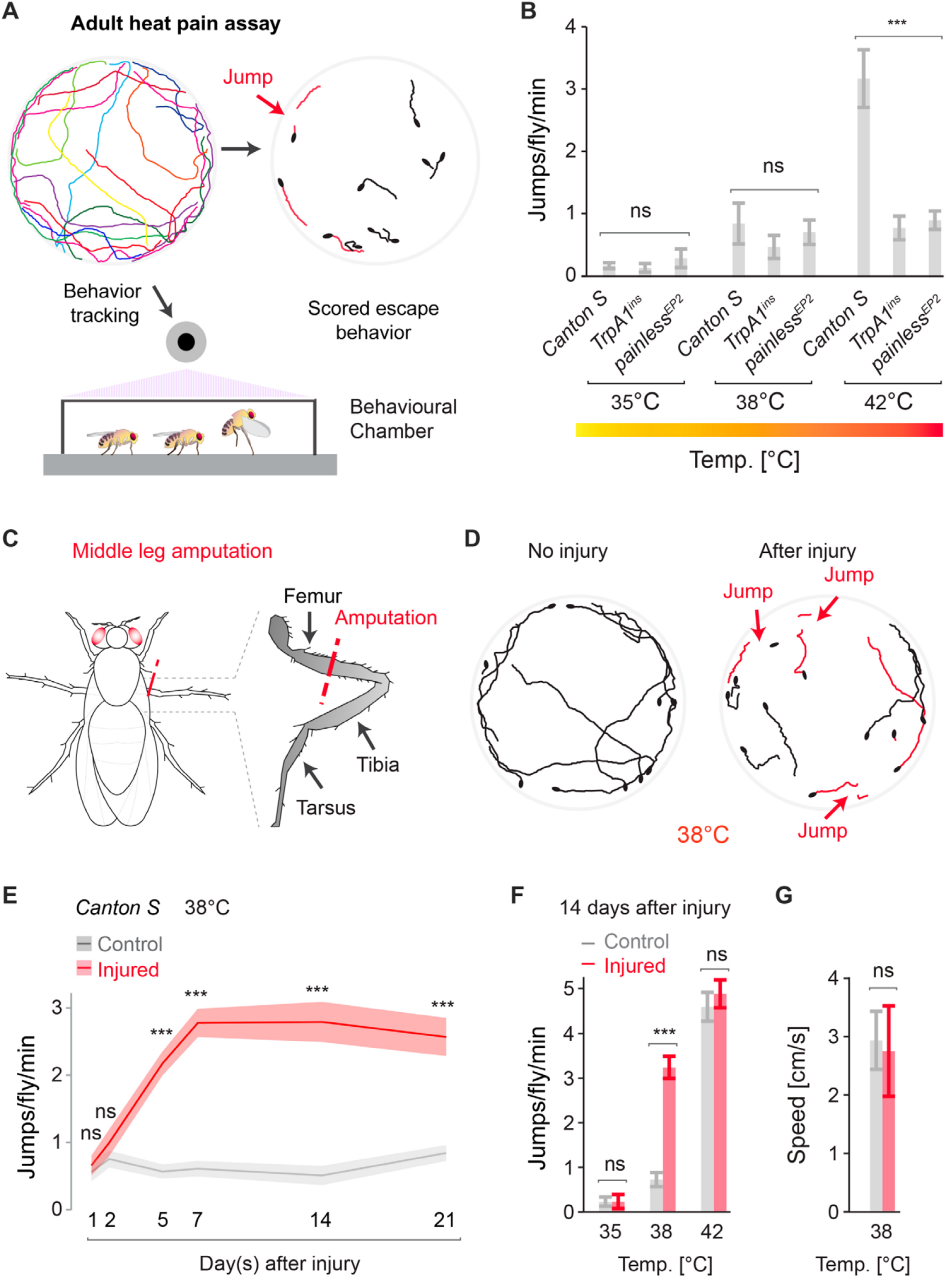
*Drosophila* exhibit thermal allodynia after injury. (**A**) Adult thermal nociception assay developed to measure nociceptive sensitization over time in the fly. (**B**) Uninjured wild-type animals exhibit escape behavior in response to temperatures of ≥42°C; this response is dependent on *painless* and *TrpA1* (*n* = 9 replicates, 10 animals per replicate). (**C**) Amputation injury used in this study. (**D**) Example tracking data from adult thermal pain assay, showing allodynia in the escape response (38°C) following injury. (**E**) Time course of allodynia response (38°C) following injury. (**F**) Dose response to temperature 14 days after injury (*n* = 9 replicates, 10 animals per replicate). (**G**) Average speed of movement for uninjured intact control or animals 7 days after injury in *Canton S* (*n* = 7 replicates, 10 animals per replicate). Data are represented as means ± SEM. ***P < 0.001; ns, not significant, two-way analysis of variance (ANOVA) followed by Tukey’s post hoc test for (B), (E), and (F) and Student’s *t* test for (G).

To investigate chronic pain in a genetically tractable invertebrate, we established a nerve injury model in the adult fruit fly. In flies, surface temperatures of ≥42°C trigger a strong nociceptive avoidance response or death within minutes (*9*). Exploiting this behavior, we developed a fly hot plate escape paradigm to investigate nociceptive thresholds (Fig. 1A). Wild-type (*Canton S*) fruit flies showed minimal escape responses when the surface was set from 25° to 38°C (Fig. 1B and movie S1). However, when animals were exposed to noxious heat (42°C), uninjured flies showed a robust nociceptive escape response with animals exhibiting ~3 escape responses per fly per minute (Fig. 1B and movie S1). Since *Drosophila TrpA* family members *TrpA1* (*9*, *25*) and *painless* (*6*, *12*) are required for acute heat nociception in larvae and adult flies, we tested whether these receptors are also involved in the acute noxious escape response. Both *TrpA1* and *painless* were necessary for acute escape behavior at the noxious (42°C) temperature (Fig. 1B).

We next injured flies and asked whether injury altered the thermal escape response profile. We amputated the right middle leg of wild-type *Canton S* flies (Fig. 1C), allowed the animals to recover, and then evaluated escape responses at different temperatures. While intact animals displayed minimal escape attempts when exposed to a 38°C surface, after amputation, flies showed significantly more escape behaviors (Fig. 1, D and E, and movie S2). This response was absent in the first 2 days after injury, first became apparent 5 days after injury and was maximum by 7 days, and persisted past 21 days (Fig. 1E and fig. S1). Injury did not significantly alter escape responses at the noxious temperature (42°C), which would be considered a hyperalgesic response, but was limited to subnoxious sensitization (38°C), consistent with thermal allodynia, where a “painful” behavioral response is elicited from an innocuous stimulus (Fig. 1F and fig. S1, A and B). Exposure to a cold surface did not elicit the same acute nociceptive escape response observed and instead slowed animal movement and then reversibly anesthetized animals without triggering escape. Injured flies showed no change in mobility after limb amputation, indicating that the phenotypes observed are not due to generalized differences in activity (Fig. 1G). Together, these data show that fruit flies exhibit allodynia and enhance escape responses following peripheral nerve injury.

### Allodynia is mediated by *TrpA1* in *ppk*+ sensory neurons

**Fig. 2:**
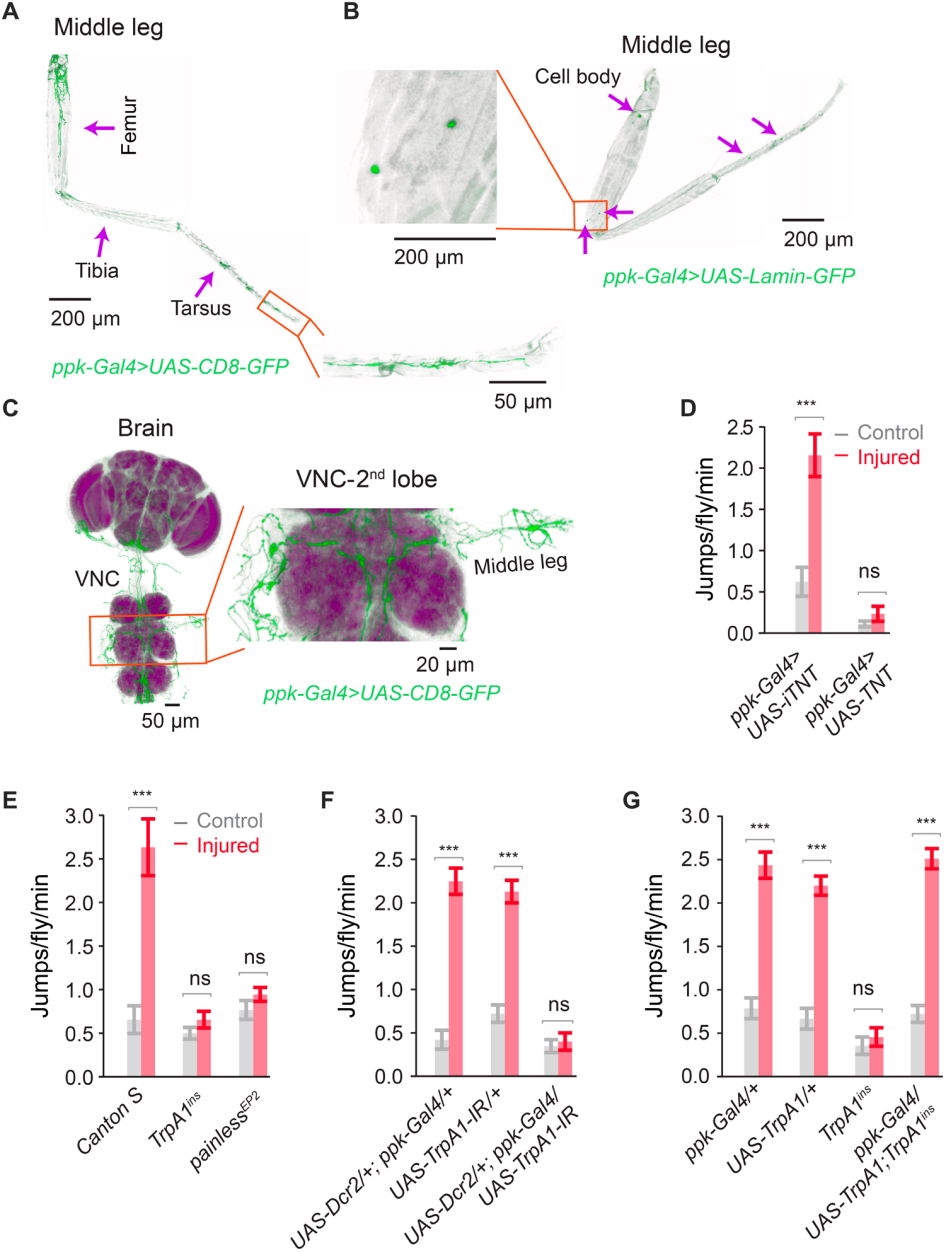
*TrpA1* is required in *ppk*+ sensory neurons for allodynia after injury. (**A**) *ppk*+ sensory neuron projections in the fly leg, labeled with CD8-GFP, *n* ≥ 7. (**B**) *ppk*+ cell bodies in the leg, labeled with Lamin-GFP, *n* ≥ 7. (**C**) *ppk*+ sensory neuron projections from the dissected leg to the VNC and brain. (**D**) Expression of active tetanus toxin (TNT) but not inactive tetanus toxin (iTNT) in *ppk*+ sensory neurons blocked all adult thermal nocifensive behavior (*n* = 9 animals, 10 animals per replicate). (**E**) *TrpA1* and *painless* mutants are resistant to thermal allodynia (38°C) (*n* = 9 replicates, 10 animals per replicate). (**F**) *TrpA1* is specifically required in *ppk*+ sensory neurons for allodynia after injury (*n* = 9 replicates, 10 animals per replicate). (**G**) Reintroduction of *TrpA1* specifically in *ppk*+ sensory neurons rescue allodynia response (*n* = 9 replicates, 10 animals per replicate). Data are represented as means ± SEM. ***P < 0.001; two-way ANOVA followed by Tukey’s post hoc test.

In larvae, *ppk*+ sensory neurons tile the body of the animal and transduce acute noxious heat responses (*26*). In the adult fly, we observed *ppk*+ neurons organized into likely sensory structures in the leg (Fig. 2A), with *ppk*+ cell bodies situated along the leg (Fig. 2B), and *ppk*+ neurons send projections both peripherally and toward the ventral nerve cord (VNC) and brain (Fig. 2C and fig. S2, A to C). When we blocked synaptic output from *ppk*+ neurons with *UAS-tetanus toxin*, animals no longer exhibited allodynia after injury (Fig. 2D) but showed otherwise comparable mobility. Moreover, while control animals exhibited a sensitized escape response to 38°C after injury, both *painless* and *TrpA1* mutant animals were completely resistant to this effect (Fig. 2E) and did not even show sensitization at 42°C (not shown). Last, driving *TrpA1* RNA interference (RNAi) in *ppk*+ sensory neurons was sufficient to block allodynia (Fig. 2F), and sensitization was completely rescued by reintroducing *TrpA1* specifically in *ppk*+ sensory neurons on a *TrpA1* mutant background (Fig. 2G). Thus, in the fly, neuropathic pain–like allodynia requires the conserved nociceptive TRP channel *TrpA1* expressed specifically in *ppk*+ nociceptive sensory neurons.

### Peripheral neuropathic injury causes allodynia via a central mechanism

**Fig. 3:**
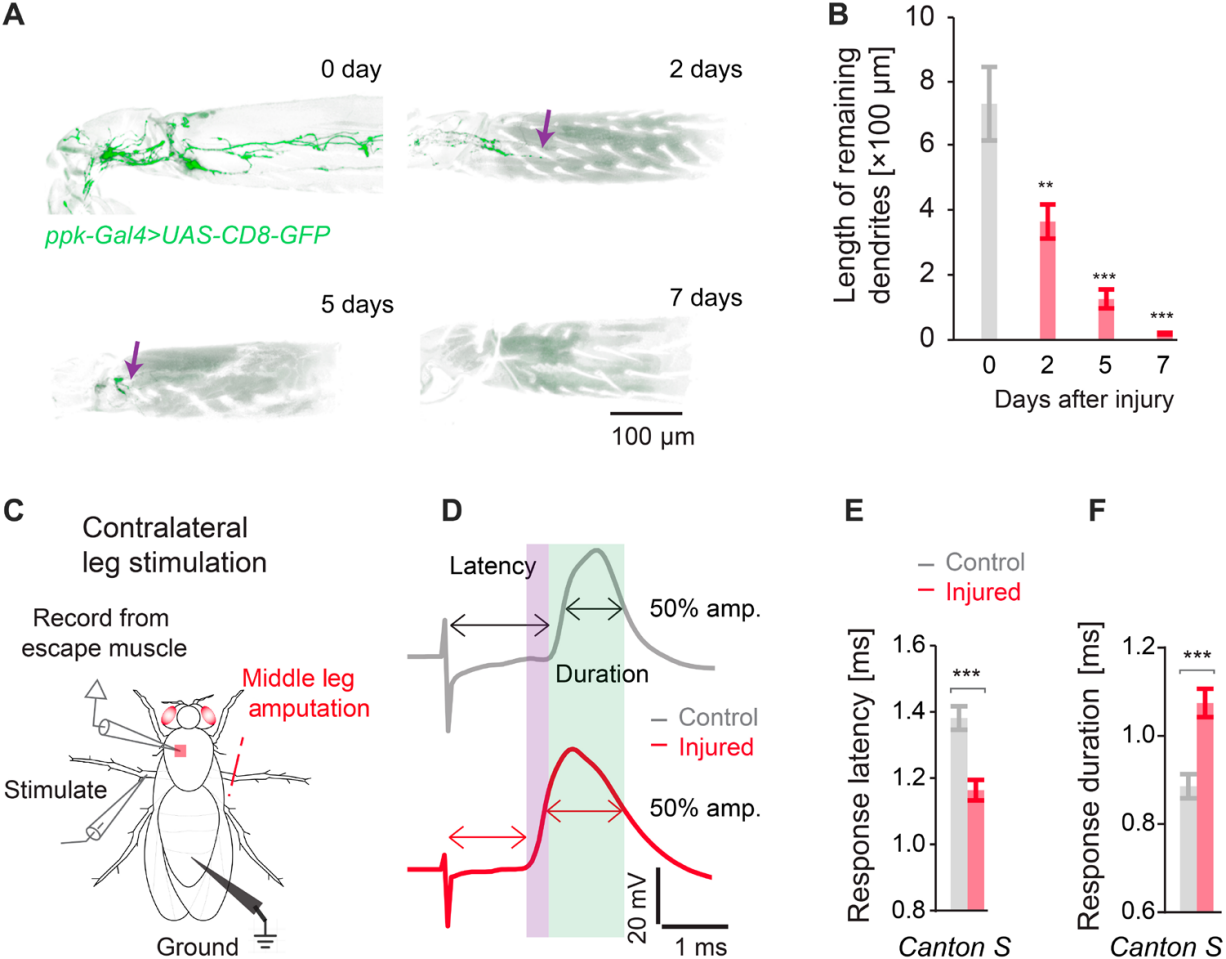
Peripheral injury leads to sensory neuropathy, central sensitization, and augmentation of the nociceptive escape circuit. (**A**) *ppk*+ sensory neuropathy is observed after leg amputation. (**B**) Quantification of sensory neuropathy (*ppk1*+ projection length) in the amputated leg over time (*n* ≥ 7). (**C**) Adult nociception electrophysiology preparation after injury. (**D** to **F**) Leg amputation results in contralateral sensitization of the escape response circuit with (D and E) increase in escape circuit velocity (velocity difference highlighted in purple) and (D and F) an increase in the duration of the escape response (injured duration highlighted in green) (*n* ≥ 9). Data are represented as means ± SEM. **P < 0.01; ***P < 0.001, two-way ANOVA followed by Tukey’s post hoc test (B) and Mann-Whitney-Wilcoxon test (E and F).

As flies exhibit a “jumping” escape response when placed on a hot (42°C) surface, and this response shows sensitization to 38°C after injury, we next investigated whether activating sensory neurons in the leg could directly trigger the escape response. We stimulated nociceptive sensilla on the middle leg of the intact fly and evaluated the escape response by intracellular recording from the dorsal longitudinal muscle (DLM), the final step in the *Drosophila* escape response circuit. Stimulation of the intact leg triggered a robust escape response (fig. S3A). The giant fiber response can occur without participation from the brain (fig. S3B). However, we found that leg stimulation leading to an escape response was not a local reflex but required higher-order brain function (fig. S3C). While amputation of the middle leg caused behavioral sensitization to innocuous heat, when we directly stimulated the injured leg, we observed no response (fig. S3D). Accordingly, we observed a gradual neuropathy of proximal *ppk*+ sensory neurons in the injured leg over 7 days (Fig. 3A, quantified in Fig. 3B), which tracked with the observed kinetics of behavioral sensitization (Fig 1E), and a similar loss of degeneration of axotomized neurons was observed after peripheral nerve transection in mammals (*27*).

Since the remaining section of the injured leg shows severe sensory neuropathy and was unresponsive to stimulation, we instead stimulated the contralateral uninjured leg of amputated flies and assessed activation of the escape response (Fig. 3C). Notably, 7 days after injury, we observed clear changes when stimulating the contralateral leg, with the overall escape response velocity occurring 0.2 ms faster (Fig. 3D, quantified in Fig. 3E) and the response duration persisting 0.2 ms longer (Fig. 3D, quantified in Fig. 3F). Together, these data show that peripheral injury leads to central changes that enhance the escape circuit response.

### Central loss of GABAergic inhibition causes neuropathic allodynia

**Fig. 4:**
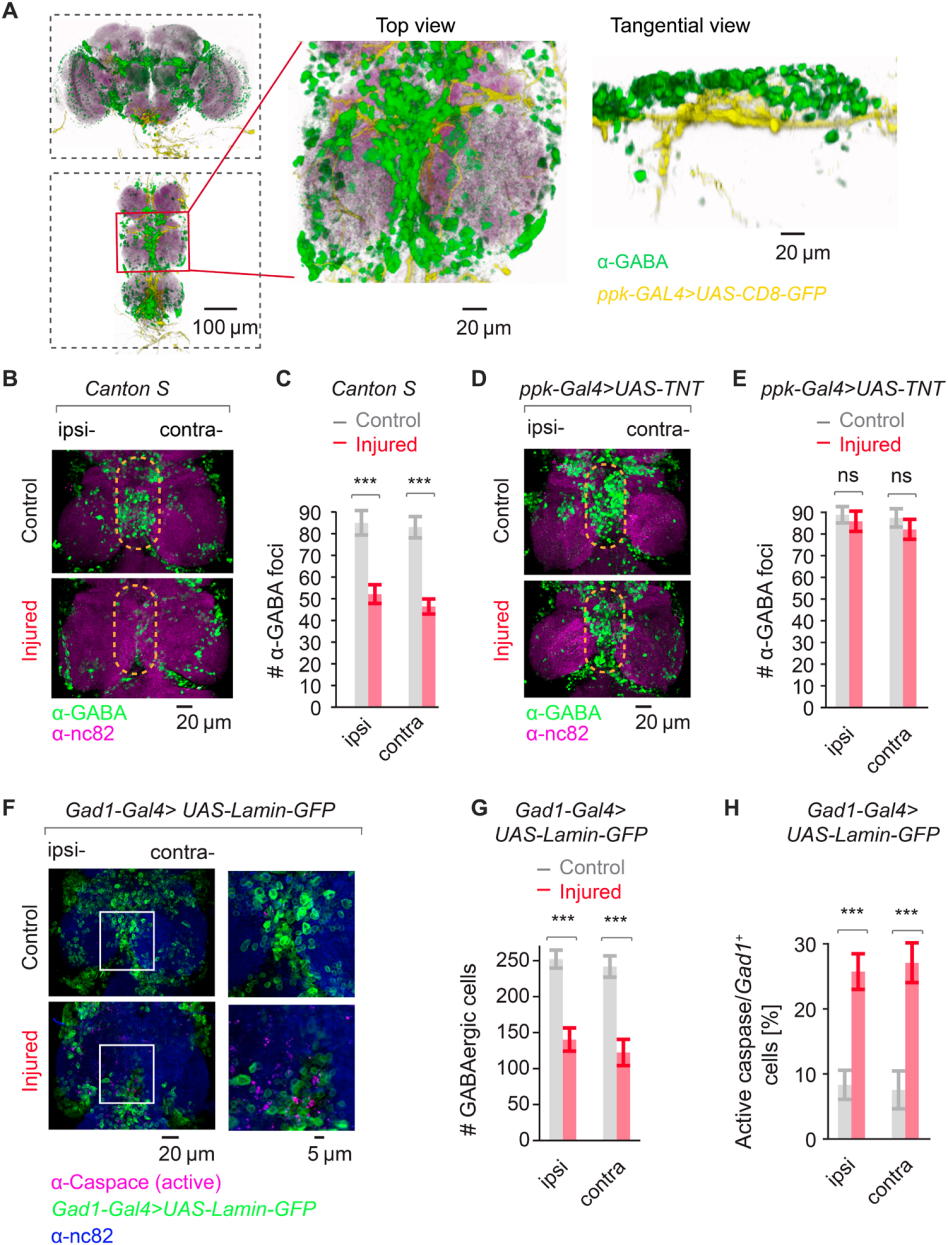
GABA gates peripheral activity; peripheral nerve injury reduces GABAergic function. (**A**) *ppk*+ sensory neuron projections to the VNC. *ppk-Gal4>UAS-CD8-GFP* is shown in yellow, anti-GABA is shown in green, nc82 contrast stain is shown in purple, showing ventral top, close-up view of anti-GABA and *ppk-Gal4>UAS-CD8-GFP* colocalization, and tangential side, close-up view of anti-GABA and *ppk-Gal4>UAS-CD8-GFP* colocalization, *n* ≥ 7. (**B**) Reduction in GABA immunoreactivity after injury of VNC stained for GABA and nc82 from uninjured and injured animals (7 days after leg amputation), showing ventral top, close-up view. (**C**) Quantification of (B), *n* ≥ 9. (**D**) Imaging of VNC GABAergic interneurons expressing *ppk-Gal4>TNT*, stained for GABA and nc82. (**E**) Quantification of (D), *n* ≥ 9. (**F**) Imaging of VNC with nuclear-labeled Lamin-GFP (*Gad1-Gal4>UAS-Lamin-GFP*) and an active caspase antibody. (**G**) Quantification of GABAergic cells in (F), *n* ≥ 9. (**H**) Quantification of active caspase/*Gad1*+ cells in (F), *n* ≥ 9. Data are represented as means ± SEM. ***P < 0.001, two-way ANOVA followed by Tukey’s post hoc test.

*ppk*+ sensory neurons project from the leg into the ventral “horn” of the *Drosophila* CNS (Fig. 2, A to C, and fig. S2, A to C). By colabeling nociceptive (*ppk*+) and GABAergic neurons, we observed a close interaction between these two populations in the VNC (Fig. 4A). Seven days after injury, we observed a marked ~40% reduction in GABA (γ-aminobutyric acid) immunoreactivity in both the ipsilateral and contralateral sections of the second VNC lobe [Fig. 4B (top view) and fig. S4A (transverse plane), quantified in Fig. 4C], with this loss primarily occurring along the VNC midline (dashed circles). A significant yet less severe reduction in GABA foci also occurred in the first and third lobes of the VNC (fig. S5, A and B); however, no difference in the number of GABA foci was observed in the brain of injured animals (fig. S5C), i.e., loss of GABA immunoreactivity was localized to the VNC. Loss of GABAergic neurons was not due to direct damage of these cells, since no GABAergic nuclei or projections were observed in the fly leg (fig. S5, D and E). Moreover, blocking synaptic output from *ppk*+ sensory neurons (*ppk-Gal4* driving *UAS-tetanus toxin*) completely prevented loss of VNC GABA foci (Fig. 4D, quantified in Fig. 4E), confirming the sensory origin and excitatory nature of this injury response.

**Fig. 5:**
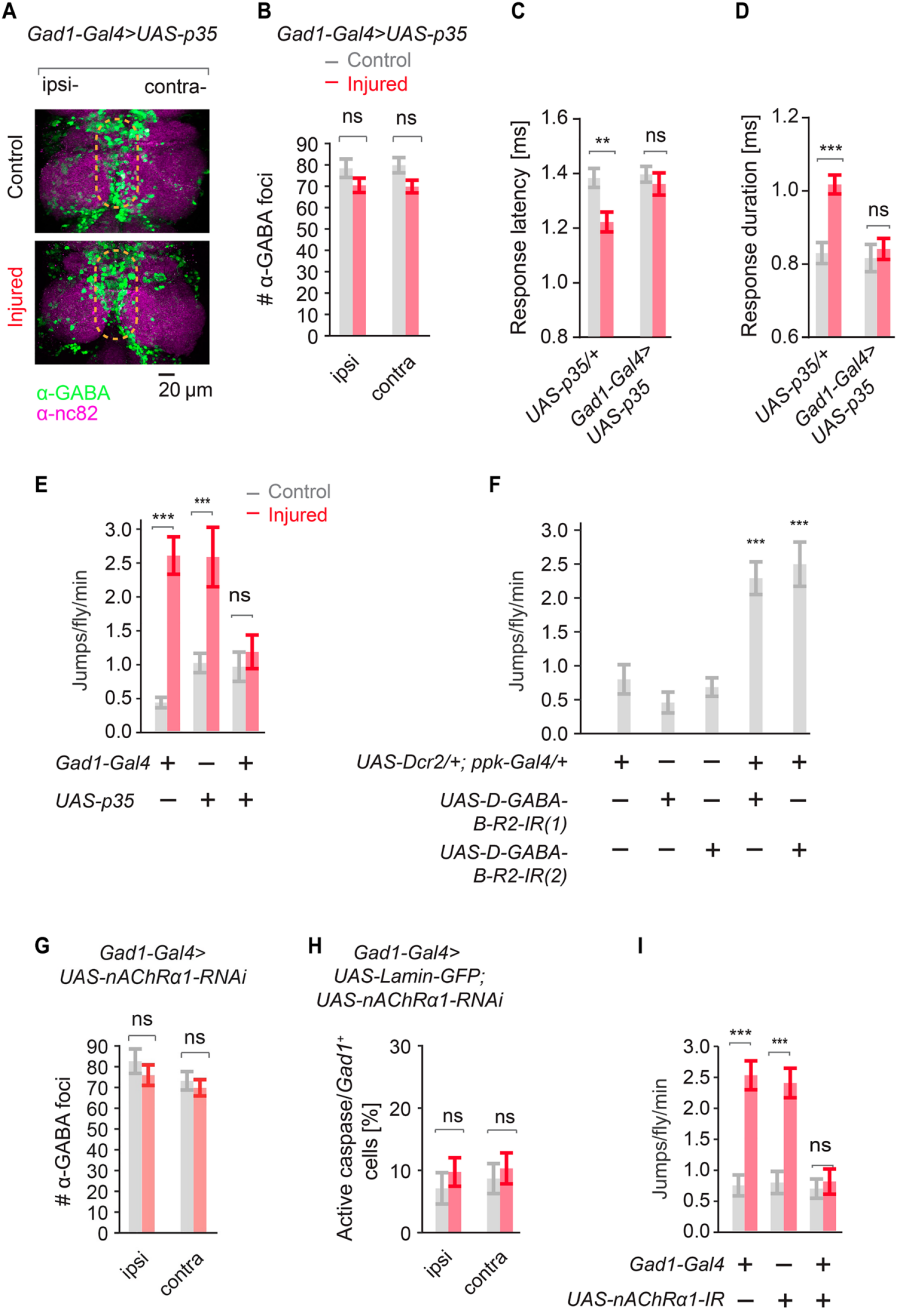
Preventing GABAergic cell death blocks changes in the nociception circuit and suppresses neuropathic allodynia. (**A**) Blocking GABAergic cell death after leg injury by *Gad1-Gal4*–driven expression of *UAS-p35* prevents GABA loss. (**B**) Quantification of (A), *n* ≥ 9. (**C** and **D**) GABAergic-specific expression of p35 (*Gad1-Gal4>UAS-p35*) rescues contralateral sensitization of the escape response circuit measured by (C) escape circuit velocity, *n* ≥ 9; (D) escape response duration, *n* ≥ 9; and (**E**) prevents neuropathic allodynia behavior (*n* = 9 replicates, 10 animals per replicate). (**F**) Nociceptive sensory neuron–specific (*ppk-Gal4*) knockdown of GABA receptor *D-GABA-B-R2* is sufficient to cause thermal allodynia (38°C) in uninjured flies (*n* ≥ 9 replicates, 10 animals per replicate). (**G**) Knockdown of nAChRα1 in GABAergic neurons (*Gad1-Gal4>nAChR*α*1-IR*) prevents GABA loss after injury. (**H**) Quantification of active caspase in GABAergic neurons (active caspase/*Gad1*+ cells) in control intact and injured flies expressing Lamin-GFP and *nAChR*α*1-IR* (*Gad1-Gal4>UAS-Lamin-GFP*; *UAS-nAChR*α*1-IR*). (**I**) Knockdown of *nAChR*α*1* in GABAergic neurons prevents neuropathic allodynia behavior (*n* ≥ 9 replicates, 10 animals per replicate). Data are represented as means ± SEM. **P < 0.01; ***P < 0.001, two-way ANOVA followed by Tukey’s post hoc test.

Since pharmacological or genetic inhibition of caspase can prevent GABA loss and suppress the generation of neuropathic pain in rodents (*28*, *29*), we assessed a role of caspase in regulating central GABA in the fly. Intact animals showed little GABAergic neurons/active caspase colabeling in the VNC (GABAergic neurons labeled by *Gad1-Gal4>>UAS-Lamin-GFP*; Fig. 4F and fig. S4B, quantified in Fig. 4H). After injury, the total number of GABAergic nuclei in the VNC was reduced by ~40% (quantified in Fig. 4G), while the number of *Gad1*/active caspase double-positive cells significantly increased (Fig. 4F, quantified in Fig. 4H). To directly test whether GABAergic cell loss is a caspase-dependent event, we drove expression of the caspase inhibitor p35 specifically in GABAergic neurons (*Gad1-Gal4>UAS-p35*). While blocking caspase did not alter the baseline number of GABA foci in the fly VNC, ectopic expression of p35 in *Gad1*+ GABAergic neurons completely blocked loss of GABA foci after nerve injury (Fig. 5A, quantified in Fig. 5B). We next tested whether blocking GABAergic cell loss had functional consequences on the overall nociceptive escape circuit in injured animals. After injury, the escape circuit showed enhanced response latency and duration in the parental control line (*UAS-p35*/*+*); however, suppressing GABAergic cell death (*Gad1-Gal4>UAS-p35*) completely blocked this effect (Fig. 5, C and D). Parental control lines exhibited neuropathic allodynia behavior after leg amputation, whereas blocking caspase-mediated GABAergic cell death completely suppressed this response (Fig. 5E). Conversely, nociceptor-specific (*ppk-Gal4*) RNAi knockdown of all GABA receptors showed targeting the metabotropic *GABA-B-R2* or the ionotropic GABA/glycine receptor subunit *Resistant to dieldrin* (*Rdl*) was sufficient to promote allodynia and enhance escape behavior in response to subnoxious temperature (38°C) in uninjured animals (Fig. 5F and fig. S6A). Together, these data show that in the fly, loss of central GABA inhibition is necessary and sufficient for thermal allodynia.

To investigate the molecular basis for how damage to sensory neurons could trigger GABAergic death, we targeted glutamatergic and cholinergic pathways in *ppk1+* neurons (*30*). While *ppk*-mediated nociception does not depend on glutamate (*ppk1-Gal4>UAS-VGlut* RNAi), targeting acetylcholine synthesis (*ppk1Gal4>UAS-ChAT* RNAi) suppressed nociception behavior (fig. S6B) (*31*). By functional RNAi screening for acetylcholine receptor subunits acting in *Gad1*+ neurons, we found that expression of the conserved *nicotinic Acetylcholine Receptor* α*1* (*nAChR*α*1*) subunit was required in GABAergic neurons for GABAergic cell death after peripheral nerve injury (Fig. 5, G and H, and fig. S6, C and D). Critically, targeting *nAChR*α*1* in GABAergic neurons was also sufficient to block the development of thermal allodynia (Fig. 5I). Thus, after injury, cholinergic nociceptive sensory neurons trigger caspase-dependent death of central inhibitory neurons via acetylcholine and *nAChR*α*1*.

**Fig. 6:**
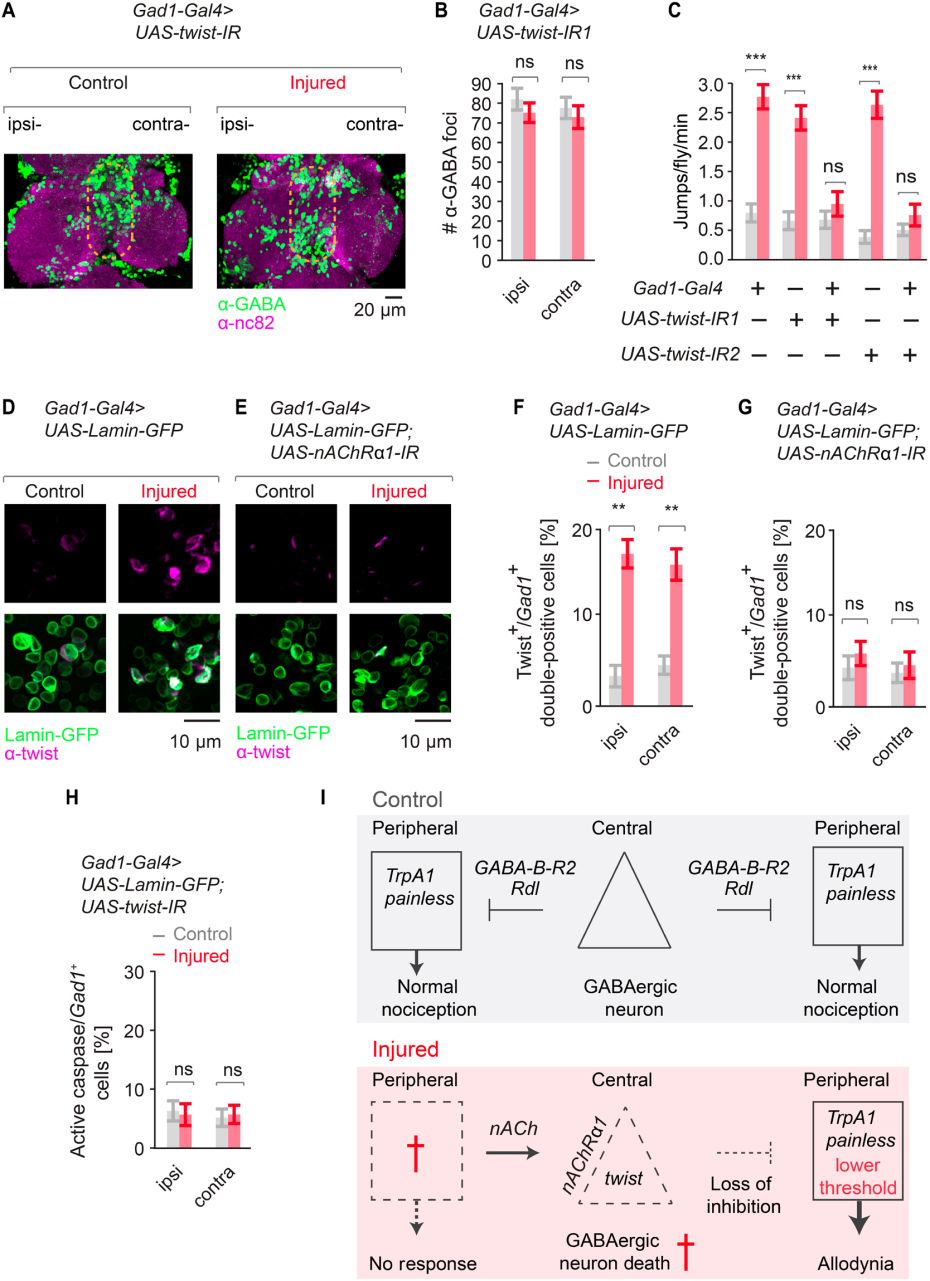
Twist mediates GABAergic excitotoxicity and central sensitization after neuropathic injury. (**A**) GABAergic (*Gad1-Gal4*) knockdown of *twist* blocks GABAergic cell death after peripheral nerve injury, *n* ≥ 9. (**B**) Quantification of (A). (**C**) *twist* is required in GABAergic neurons for neuropathic allodynia to develop after nerve injury (*n* ≥ 9 replicates, 10 animals per replicate). (**D**) Leg amputation results in the induction of Twist protein in GABAergic neurons of the VNC. (**E**) GABAergic (*Gad1-Gal4*) knockdown of *nAChR* prevents the induction of Twist protein caused by leg injury. (**F**) Quantification of (D), *n* ≥ 9. (**G**) Quantification of (E), *n* ≥ 9. (**H**) GABAergic (*Gad1-Gal4*) knockdown of *twist* prevents increased active caspase/*Gad1*+ cells, *n* ≥ 9. (**I**) Model for how injury leads to thermal allodynia in the fly. Data are represented as means ± SEM. ***P < 0.001; **P < 0.01; two-way ANOVA followed by Tukey’s post hoc test.

To find new regulators of cholinergic excitotoxicity, we performed a whole-genome screen for neural protection from nicotine toxicity and isolated hundreds of neural excitotoxicity resistance genes. We further tested the top excitotoxic death genes for a role in the fly neuropathic response and found that the basic helix-loop-helix transcription factor *twist* was critical for loss of VNC GABAergic neurons following nerve injury (Fig. 6A, quantified in Fig. 6B). GABA-specific targeting of *twist* also protected animals from developing neuropathic allodynia (Fig. 6C and fig. S6E). Accordingly, while minimal Twist protein expression was observed in VNC GABAergic neurons from uninjured animals, after neuropathic injury, we observed a strong up-regulation of Twist in GABA-positive cells (Fig. 6D, quantified in Fig. 6F). Moreover, Twist expression occurred downstream of *nAChR*α*1* signaling, since *Gad1Gal4>nAChR*α*1* RNAi knockdown animals failed to up-regulate Twist after peripheral injury (Fig. 6E, quantified in Fig. 6G). Last, Twist was acting upstream of caspase, since RNAi targeting of *twist* in GABAergic neurons suppressed caspase activation (Fig. 6H). Together, we find that peripheral nerve injury leads to *nAChR*α*1-*mediated *twist*-dependent central GABAergic interneuron apoptosis, and this leads to central disinhibition, neuropathic allodynia, and nociceptive hypervigilance in the fly.

## DISCUSSION

In this study, we show that *D. melanogaster* can enter a neuropathic pain–like state after injury. By combining behavioral assessment of neuropathic allodynia with genetic and electrophysiological approaches, we performed a systematic molecular dissection of this phenomenon from first principles. We identified a novel neuropathic cascade emanating from the injured peripheral neuron and triggering *nAChR*α*1*/*twist*/*caspase*-mediated excitotoxic death of central GABAergic inhibitory neurons, culminating in neuropathic allodynia (Fig. 6I). Together, these data highlight a previously unknown neuropathic injury response program that promotes heightened sensory vigilance and an augmented escape response changes that may help promote survival in dangerous environments.

We found that damaging the fly leg triggered heat allodynia, and this occurred through a neuropathic mechanism. After the initial damage, the remaining sensory neurons in the leg exhibited peripheral degeneration as allodynia developed, and in human pain patients, peripheral neuropathy is a common cause of neuropathic pain (*18*). While our study here is the first description of long-lasting chronic pain in the fly, there are numerous studies describing models of peripheral neuropathy in larvae or adult flies. For example, exposure to chemotherapeutics such as taxol causes both painful and painless peripheral neuropathies in both patients (*32*), and similar painful sensory neuropathies develop in fly chemotherapy-treated larvae (*33*–*36*). Of note, similar to our observations here, painful peripheral neuropathy in response to the chemotherapy agent vinblastine also requires *TrpA1* both in flies and mice, suggesting that the underlying mechanism of sensitization in response to different injuries may show some conservation (*35*). While long-lasting chronic pain is not possible to evaluate in *Drosophila* larval, adult flies also exhibit a marked impairment of motor function in response to the chemotherapeutic cisplatin (*37*). This response is likely a result of a peripheral neuropathy and may involve pain; however, because of the motor dysfunction observed directly, evaluating pain in this system is difficult. Together, there are now multiple available systems to dissect peripheral neuropathies and neuropathic pain in the fly, and *Drosophila* genetics coupled with mammalian validation will be a powerful system to investigate the molecular mechanisms leading to both painful and painless neuropathies.

It is not completely clear why chronic pain develops after injury. In rodents, pain sensitivities are modulated by GABAergic central inhibition in the spinal cord (*38*), and a loss of GABA-dependent inhibitory postsynaptic potential (PSP) can be observed after some forms of nerve injury (*39*). This is accompanied by a decrease in GABA release and reduced expression of the GABA synthesizing enzymes (*40*). Loss of central GABA has also been observed in humans suffering from chronic neuropathic pain (*41*). Moreover, genetic ablation of spinal inhibitory populations is sufficient to cause allodynia in mice (*42*, *43*). Transplanting GABAergic interneurons into the spinal cord of mice suffering from neuropathic pain can reverse mechanical allodynia (*44*). Mechanistically, the reduction in spinal GABA may be linked to GABAergic Ca^2+^ influx (*45*), reactive oxygen species (*46*), and apoptosis of GABAergic interneurons (*28*, *29*). While systemic antioxidant treatment or intrathecal delivery of a specific caspase inhibitor can prevent GABAergic cell death and attenuate neuropathic pain behavior (*28*, *46*), definitive genetic proof that death of GABAergic interneurons cause neuropathic sensitization was, until now, lacking. In our system, we observed GABAergic apoptosis after injury, and this led to changes in the nociceptive escape circuit physiology and behavioral sensitization to innocuous temperature, all of which are hallmarks of human neuropathic pain. Through precise genetic manipulation, we could inhibit caspase-dependent cell death specifically in GABAergic interneurons in the VNC, and this completely blocked development of the neuropathic pain–like state in flies. Of note, while we assume that contralateral hypersensitization was caused via excitotoxic acetylcholine release from the injured leg, it is also possible that injury causes a compensatory increase in activity of the contralateral leg, and this increased contralateral movement then drives central GABAergic cell death. Regardless, dysregulation of central GABA tone appears to be a core conserved mechanism critical for neuropathic pain disease across diverse species.

Sensitization after injury is not strictly a vertebrate phenomenon. For example, *Drosophila* larvae show a transient allodynia after ultraviolet exposure (*16*), and injury can also lead to nociceptive sensitization in the marine mollusk *Aplysia* (*19*–*22*, *47*) or the caterpillar *Manduca sexta* (*48*). Alternatively, damaging the fin or arm of the squid *D. pealei* causes long-lasting sensitization (*23*), and injured animals also acquire enhanced escape responses to natural predators. Together, these data suggest that neuropathic sensitization may induce a heightened state of vigilance that confers a survival advantage during predation (*1*, *24*). While contralateral sensitization was also reported in the squid and would presumably occur via a central mechanism (*23*) such as loss of inhibitory tone, this remains to be investigated. Regardless, these data fit nicely with the fly neuropathic response described here. We observed global augmentation of the escape circuit and contralateral sensitization after nerve injury, and in the fly, these changes are mediated through an irreversible loss of central inhibition. The sensitization we observed is a bona fide neuropathic response, since synaptic silencing of the injured sensory neurons completely blocks central disinhibition. Together, these data suggest that after serious injury, invertebrates use a neuropathic response to acquire a heightened state of vigilance, and this response is a common protective mechanism in invertebrates and may be the evolutionary precursor for maladaptive neuropathic pain in humans.

We found the basic helix-loop-helix transcription factor *twist* critical for GABAergic cell death after peripheral nerve injury. *Twist* was originally identified as a regulator of dorsal-ventral patterning in the developing fly (*49*, *50*). While *twist* has been characterized for its role in mesoderm development and mammalian epithelial-mesenchymal transition during metastasis (*51*), developmental defects in peripheral nervous system and CNS have also been reported in *twist* mutant flies (*52*) and mice (*53*), and virtually, nothing is known about *twist* function in adult neurons. We find that *twist* is not required for GABAergic cell development but is an essential component of the GABAergic excitotoxic response leading to the loss of central GABA and neuropathic sensitization. The role of *twist* in regulating cell death is unclear. In cell culture, human *twist* can suppress p53-dependent cell death (*54*–*56*); however, in vivo expression of *Drosophila twist* is sufficient to drive apoptosis in the wing disc (*57*). Our data support a role of *twist* in promoting excitotoxic cell death after nerve injury. We found that GABAergic cell death was also dependent on caspase activation; however, the precise molecular interactions downstream of the *nAChR*α*1/twist* leading to caspase activation and GABAergic cell death remain to be investigated.

In many ways, chronic pain states exhibit similarities with experience-driven learning and memory (*19*, *58*). For example, repeated stimulation of primary sensory afferents is sufficient to induce a state of postsynaptic long-term potentiation in the mammalian spinal cord (*59*–*61*), and the capacity for a similar nociceptive sensitization has been reported in various invertebrate systems (*19*, *23*, *24*, *62*, *63*). Peripheral stimulation and the resulting central synaptic changes are key molecular events leading to nociceptive sensitization during some forms of chronic pain (*64*). In the adult fly, we also observed experience-driven plasticity of nociception circuitry, including an overall increase in the velocity of impulse transmission from sensory neuron to escape muscles, increased persistence of nociceptive response after stimulation, and increased probability of response. These data are compatible with synaptic reinforcement within the dedicated nociception circuit, or alternatively after injury, nociceptive responses could be rerouted through an alternate escape circuit with different response properties (*65*). Whether the observed change in escape circuit properties involve synaptic plasticity similar to spinal long-term potentiation in mammals remains to be addressed; however, our genetic evidence places GABAergic cell death as a critical upstream event leading to global changes both in circuitry and neuropathic sensitization in the fly.

Here, we describe the first chronic pain paradigm in the fruit fly. This system, when coupled with genetic or pharmacological intervention, can rapidly inform on neuropathic mechanisms leading to central disinhibition and allodynia. Our findings here confirm that sensory sensitization after nerve injury is conserved across phyla, and the loss of central inhibition observed in the fly is consistent with some rodent and human neuropathic pain states. Our studies are in line with previous work (*24*) that suggests that neuropathic responses may have originally been beneficial, and the heightened state of vigilance injured invertebrates exhibit may have provided an evolutionary protective advantage after serious injury. Thus, while acute nociception first evolved more than ~500 million years ago, neuropathic pain also appears to be an ancient and conserved response.

## MATERIALS AND METHODS

### *Drosophila* stocks

Flies were reared on a standard corn meal, yeast, and sucrose agar medium at 25°C under a 12-hour/12-hour light/dark cycle. *Canton S* (BDSC 64349), *painless* [*EP(2)2451*] (BDSC 27895), *ppk-Gal4* (BDSC 32078), *UAS-CD8-GFP* (BDSC 5130), *UAS-Dcr2*, *UAS-tetanus toxin* (active, BDSC 28838 and inactive BDSC 28839), *UAS-p35* (BDSC 5072), and *UAS-Lamin-GFP* (BDSC 7376) flies were obtained from the Bloomington Drosophila Stock Center (BDSC) library. *w1118* (VDRC 60000), *UAS-TrpA1-RNAi* (VDRC 37249), *UAS-RDL-RNAi* (VDRC 41101), *UAS-GRD-RNAi* (VDRC 5329), *UAS-D-GABA-B-R1-RNAi* (VDRC 101440), *UAS-D-GABA-B-R2-RNAi* (VDRC 110268 and VDRC 1785), *UAS-D-GABA-B-R3-RNAi* (VDRC 50176), *UAS-LCCH3-RNAi* (VDRC 37408), *UAS-Vglut-RNAi* (BDSC 27538), *UAS-ChAT-RNAi* (BDSC 25856), *UAS-nAChR*α*1-RNAi* (VDRC 48159), and *UAS-twist-RNAi* (VDRC 37091 and VDRC 37092) flies were obtained from the Vienna Drosophila Resource Center (VDRC) RNAi library (*66*). *UAS-TrpA1* and *TrpA1^ins^* flies were from P. Garrity, and *Gad1-Gal4* flies were from H. Bellen.

### Adult thermal nociception assay system

The adult thermal nociception assay system consists of transparent polystyrene test chambers (0.3-cm height, 5.5-cm diameter clear plastic lid), a variable heat element [model AHP-1200DCP, part number 9-34KB-1-0A1, of ThermoElectric Cooling America (TECA) Corp., IL], a movie recording setup, and behavior analysis software. Movies were recorded with a single camera from top (Canon EOS, 700D, 18- to 55-mm lens).

### Fly injury model

The right middle leg was amputated at the femur segment using vannas scissors (World Precision Instruments). Flies were 7 days old when the leg was amputated and tested 1, 2, 5, 7, 14, or 21 days later. Each set of 10 flies was lightly anesthetized on ice before being placed in a behavioral chamber. Surface was initially set at 25°C. Flies were allowed to acclimate to the test chamber, and then baseline 25°C responses were recorded. Surface temperature was held at 25°C for 2 min and then raised to 30°C for 2 min, then similarly to 35°C for 2 min, 38°C for 2 min, and lastly at 42°C for 1 min. A video recording camera set at 29-fps images/s and positioned above the apparatus was used to record observations of flies. Jumping behavior was scored manually blind to the treatment using recorded videos. Speed of movement was measured using Ctrax software that track individual flies. For each experiment, three batches of 10 flies were tested, and then results were repeated with three independent groups (*n* = 9 replicates). Statistical analysis was performed using *t* test for single comparisons and ANOVA, followed by a post hoc Tukey’s test for multiple comparisons.

### Electrophysiological recordings

Flies were anesthetized using ice and anchored to a wax support ventral side down. Two stimulating electrodes made of tungsten connected to a stimulator (Constant Voltage Isolated Stimulator, model DS2A-Mk.II, Digitimer) were placed into both eyes to activate the giant fiber system (GFS). Similarly, two tungsten stimulating electrodes were also placed in the middle of femur segment of the right (ipsilateral) or left (contralateral) leg to activate nociceptive GFS escape through the leg. For GFS through the eye, flies were given 20 single stimuli with a maximum stimulation intensity smaller than 15 V. For leg stimulation nociceptive escape, the maximum stimulation intensity was less than 60 V. For all experiments, stimulation duration was kept constantly at 10 μs. A tungsten ground electrode was placed into the fly abdomen. A tungsten recording electrode, sharpened in sodium hydroxide 5M (with a bench-top power supply, PSU 130-LASCAR), was placed into the left backside of the fly at the DLM fiber to record the PSPs. PSPs of at least nine flies for each group were recorded using Microelectrode AC Amplifier, Model 1800(A-M System) filtered at 0.5 kHz and digitized at 1 kHz. PSPs were analyzed using AxoGraph software (AxoGraph Scientific, Berkeley, CA). To determine whether the response measured by stimulating the leg was mediated by the CNS, a similar setup for recordings was used, with the head of the fly removed. Mann-Whitney rank sum test was used to determine differences in response latency and duration.

### Immunohistochemistry studies and imaging

Immunofluorescence on fly brains and VNCs was performed as described (*67*). Anti-GABA (A2052, Sigma-Aldrich) was used at a dilution of 1:500, nc82 antibody (Developmental Studies Hybridoma Bank) at a dilution of 1:75, cleaved caspase-3 antibody (Asp175, Cell Signaling Technology) at a dilution of 1:500, and anti-Twist (GTX127310, GeneTex) at a dilution of 1:500. Secondary antibodies (Alexa Fluor 488, Alexa Fluor 555, and Alexa Fluor 647 from Thermo Fisher Scientific) were used at dilutions of 1:500. Confocal sections were acquired using a Leica DMI 6000 SP8 confocal microscope with 40× numerical aperture (NA) 1.30 oil objective at 0.6-μm intervals and with 63× NA 1.4 oil objective at 0.34 μm. Top-view pictures were made by performing maximum projections of image stacks in ImageJ (National Institutes of Health; http://rsbweb.nih.gov/ij/), and tangential side-view images were made by using ImageJ and Leica Application Suite X (LASX) software. GABA foci and *Gad1-LaminGFP*–positive cells were quantified using three-dimensional object counter function in ImageJ. Leg imaging was performed at 16×/0.5 multi-immersion (IMM) objective at 2.34-μm intervals, and tarsus segment imaging was acquired at 40× oil objective at 0.6-μm intervals, of the same confocal microscope. Neuropathy of *ppk+* neurons in the leg was assessed by measuring dendritic length retained in the leg using ImageJ.

### Larval nociception behavior assay

Larval nociception behavior assay was performed as described (*12*). Briefly, third instar larvae were transferred to a petri dish (10 cm) containing a thin film of distilled water. The larvae were allowed at least 10 min to habituate to the plate. A thermal heat probe (46°C) was touched to abdominal segments A4 to A6, and the response time was recorded as the time taken for the characteristic barrel roll response to occur. At least 60 animals were tested for each genotype.

## Supplementary Material

http://advances.sciencemag.org/cgi/content/full/5/7/eaaw4099/DC1

Download PDF

Movie S1

Movie S2

Table S2

## SUPPLEMENTARY MATERIALS

Supplementary material for this article is available at http://advances.sciencemag.org/cgi/content/full/5/7/eaaw4099/DC1 Fig. S1. Injury causes persistent allodynia. Fig. S2. *ppk*+ sensory neuron projections to the VNC and brain. Fig. S3. Electrophysiological properties of the nociceptive escape circuit. Fig. S4. Peripheral injury causes a loss of GABAergic interneurons. Fig. S5. Peripheral injury causes reduction in GABA in the VNC but not the brain. Fig. S6. Knockdown of *Grd*, *GABA-B-R1*, or *GABA-B-R3* does not cause allodynia in uninjured flies, cholinergic output from *ppk+* neurons mediates acute nociception behavior, and Twist is important for GABA loss after injury and mediates heat allodynia. Movie S1. Uninjured wild-type animals exhibit escape behavior in response to temperatures of ≥42°C. Movie S2. Peripheral injury causes increase in thermal allodynia in wild-type flies. Table S1. List of antibodies used in immunochemical experiments. Table S2. Detailed data of behavioral, immunochemical, and electrophysiological experiments.
