# Supplementary material for: Nerve injury drives a heightened state of vigilance and neuropathic sensitization in Drosophila
Source: Sci Adv. 2019 Jul 10;5(7):eaaw4099. doi: 10.1126/sciadv.aaw4099 (PMC6620091; doi:10.1126/sciadv.aaw4099)
Supplement: http://advances.sciencemag.org/cgi/content/full/5/7/eaaw4099/DC1 [file supp_5_7_eaaw4099__index.html]

Science Advances | Science AdvancesAAASSearchScience AdvancesMenu

## Supplementary Materials

**The PDF file includes:**

- Fig. S1. Injury causes persistent allodynia.
- Fig. S2. *ppk*+ sensory neuron projections to the VNC and brain.
- Fig. S3. Electrophysiological properties of the nociceptive escape circuit.
- Fig. S4. Peripheral injury causes a loss of GABAergic interneurons.
- Fig. S5. Peripheral injury causes reduction in GABA in the VNC but not the brain.
- Fig. S6. Knockdown of *Grd*, *GABA-B-R1*, or *GABA-B-R3* does not cause allodynia in uninjured flies, cholinergic output from *ppk+* neurons mediates acute nociception behavior, and Twist is important for GABA loss after injury and mediates heat allodynia.
- Table S1. List of antibodies used in immunochemical experiments.
- Legends for movies S1 and S2
- Legend for table S2

Download PDF

**Other Supplementary Material for this manuscript includes the following:**

- Movie S1 (.mp4 format). Uninjured wild-type animals exhibit escape behavior in response to temperatures of ≥42°C.
- Movie S2 (.mp4 format). Peripheral injury causes increase in thermal allodynia in wild-type flies.
- Table S2 (Microsoft Excel format). Detailed data of behavioral, immunochemical, and electrophysiological experiments.

**Files in this Data Supplement:**

- Adobe PDF - aaw4099\_SM.pdf
