# Supplementary material for: Nerve injury drives a heightened state of vigilance and neuropathic sensitization in Drosophila
Source: Sci Adv. 2019 Jul 10;5(7):eaaw4099. doi: 10.1126/sciadv.aaw4099 (PMC6620091; doi:10.1126/sciadv.aaw4099)
Supplement: Download PDF [file aaw4099_SM.pdf]

Science Advances
AAAS

advances.sciencemag.org/cgi/content/full/5/7/eaaw4099/DC1

Supplementary Materials for

Nerve injury drives a heightened state of vigilance and neuropathic sensitization in *Drosophila*

Thang M. Khuong, Qiao-Ping Wang, John Manion, Lisa J. Oyston, Man-Tat Lau, Harry Towler, Yong Qi Lin, G. Gregory Neely*

*Corresponding author. Email: greg.neely@sydney.edu.au

Published 10 July 2019, *Sci. Adv.* **5**, eaaw4099 (2019)
DOI: 10.1126/sciadv.aaw4099

The PDF file includes:

Fig. S1. Injury causes persistent allodynia.
Fig. S2. *ppk*+ sensory neuron projections to the VNC and brain.
Fig. S3. Electrophysiological properties of the nociceptive escape circuit.
Fig. S4. Peripheral injury causes a loss of GABAergic interneurons.
Fig. S5. Peripheral injury causes reduction in GABA in the VNC but not the brain.
Fig. S6. Knockdown of *Grd*, *GABA-B-R1*, or *GABA-B-R3* does not cause allodynia in uninjured flies, cholinergic output from *ppk*+ neurons mediates acute nociception behavior, and Twist is important for GABA loss after injury and mediates heat allodynia.
Table S1. List of antibodies used in immunochemical experiments.
Legends for movies S1 and S2
Legend for table S2

Other Supplementary Material for this manuscript includes the following:

(available at advances.sciencemag.org/cgi/content/full/5/7/eaaw4099/DC1)

Movie S1 (.mp4 format). Uninjured wild-type animals exhibit escape behavior in response to temperatures of ≥42°C.
Movie S2 (.mp4 format). Peripheral injury causes increase in thermal allodynia in wild-type flies.
Table S2 (Microsoft Excel format). Detailed data of behavioral, immunochemical, and electrophysiological experiments.

SUPPLEMENTARY MATERIALS

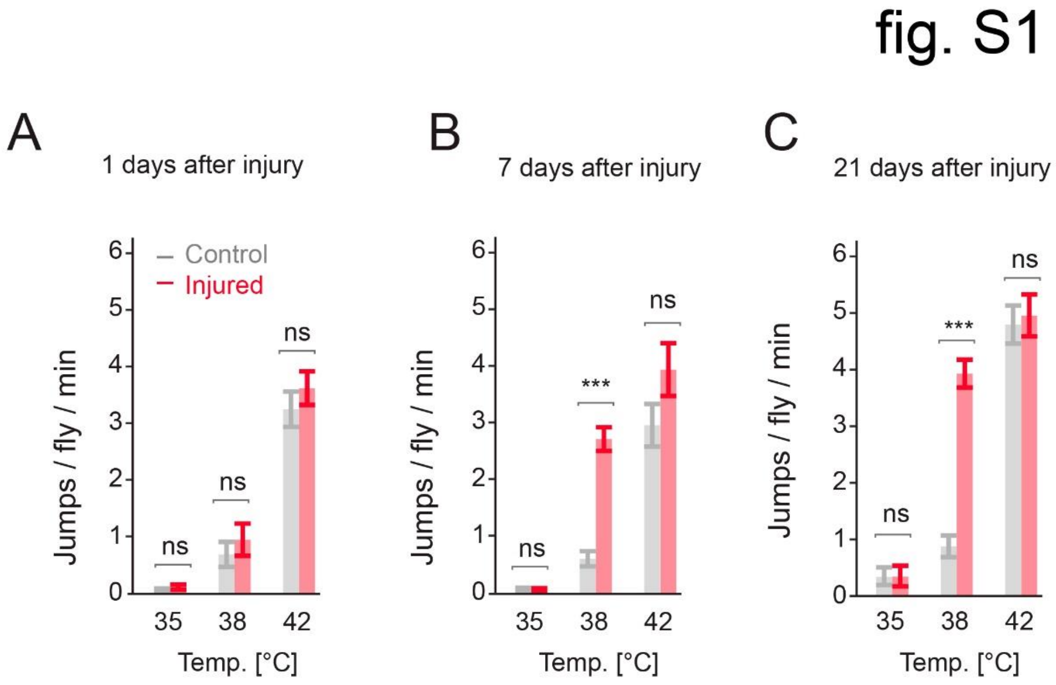

Fig. S1. Injury causes persistent allodynia. (A to C) Time course and dose-response to temperature after injury, (n=9 replicates, 10 animals per replicate). Data are represented as mean ± SEM. ****p*<0.001; ns, not significant, student's *t*-test.

fig. S2

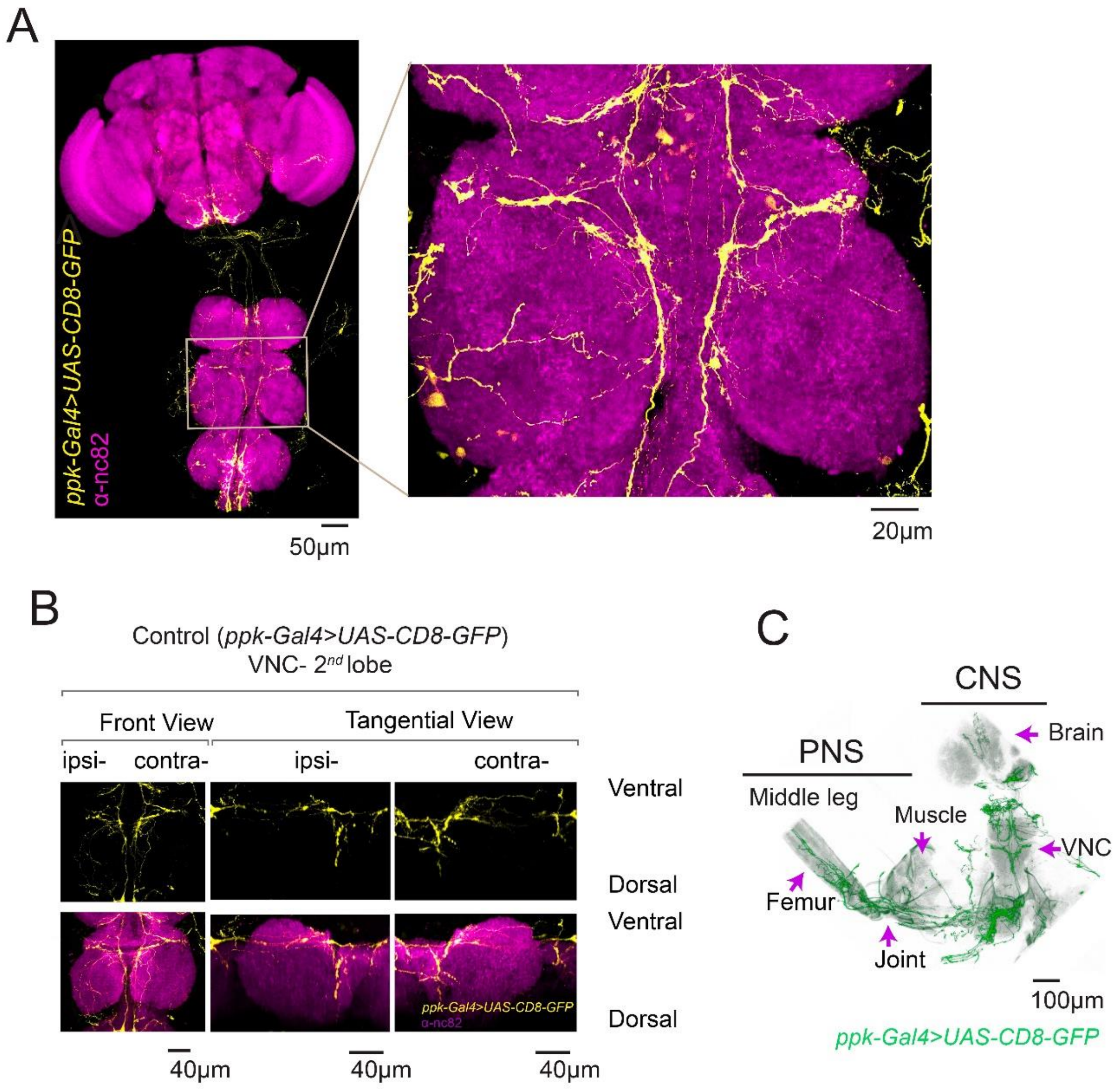

Fig. S2. *ppk*+ sensory neuron projections to the VNC and brain. (A and B) Brain and attached VNC of flies expressing CD8-GFP driven by *ppk-Gal4* (*ppk-Gal4>UAS-CD8-GFP)* (yellow) and co-stained for nc82 (magenta) with (A) ventral top view and (B) ventral top and tangential side view of 2nd lobe of VNC (bottom panels); n=6. (C). Connected brain, attached VNC, and part of femur segment of *ppk-Gal4>UAS-CD8-GFP* flies (GFP is green), n=5.

fig. S3

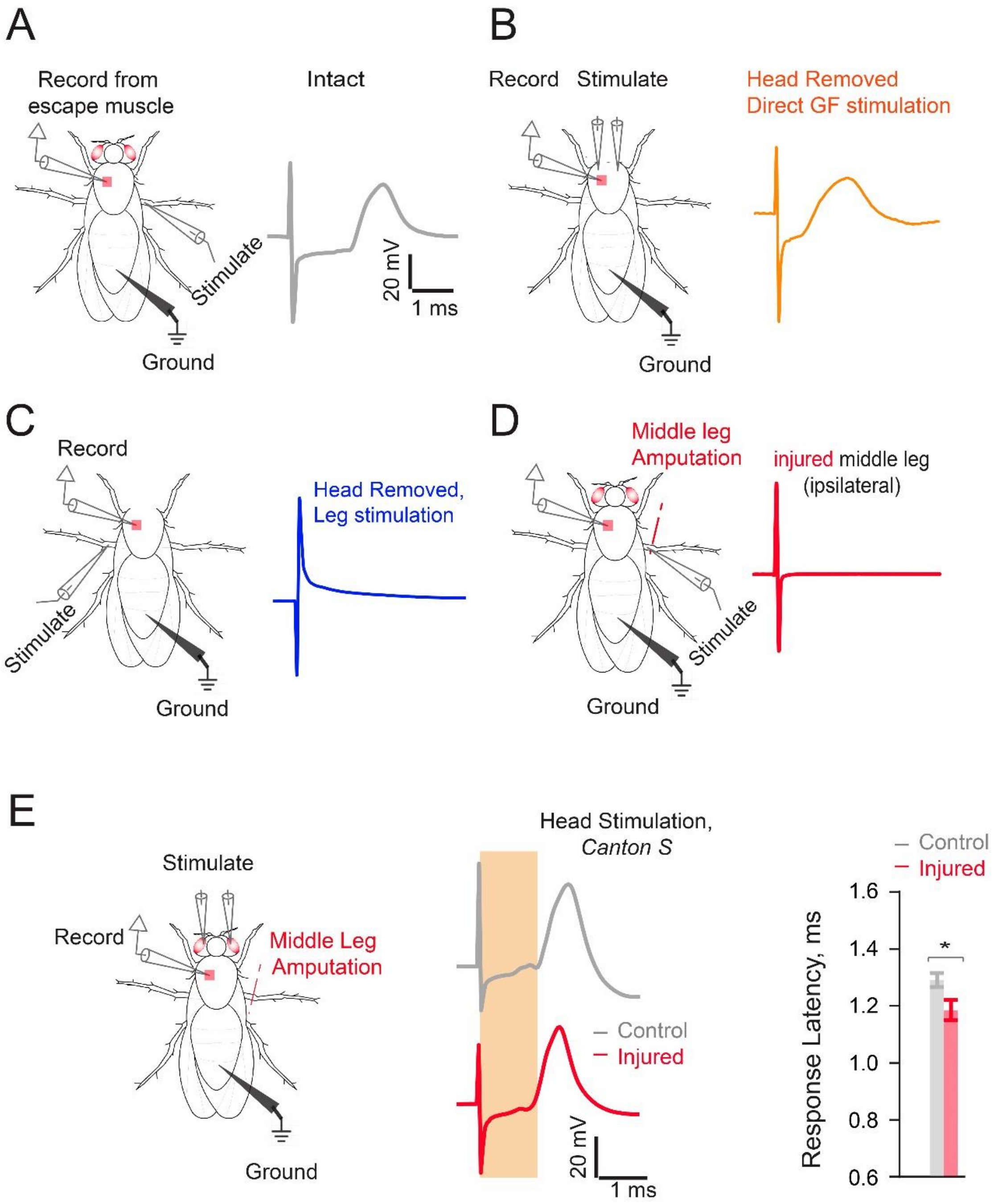

A
Record from
escape muscle
Intact
Stimulate
20 mV
1 ms
Ground
B
Record
Stimulate
Head Removed
Direct GF stimulation
Ground
C
Record
Head Removed,
Leg stimulation
Stimulate
Ground
D
Middle leg
Amputation
injured middle leg
(ipsilateral)
Stimulate
Ground
E
Stimulate
Record
Middle Leg
Amputation
Ground
Head Stimulation,
Canton S
Control
Injured
20 mV
1 ms
Control
Injured
Response Latency, ms
1.6
1.4
1.2
1.0
0.8
0.6
*

Fig. S3. Electrophysiological properties of the nociceptive escape circuit. (A) Electrophysiological recordings from DLM, the output of giant fibre system in intact animals ($n \geq 7$). (B to D) The giant fibre response requires higher order brain function. (B) Direct stimulation from giant fibre neuron in flies with the head removed, ($n \geq 7$). (C) Stimulation from the intact middle leg in flies with the head removed, ($n \geq 7$). (D) Stimulation of the injured leg 7 days after amputation, ($n \geq 7$). (E) Descending stimulation from the head of injured flies still shows decreased response latency, ($n \geq 9$). Data are represented as mean ± SEM. *$p < 0.05$, Mann-Whitney-Wilcoxon test.

fig. S4

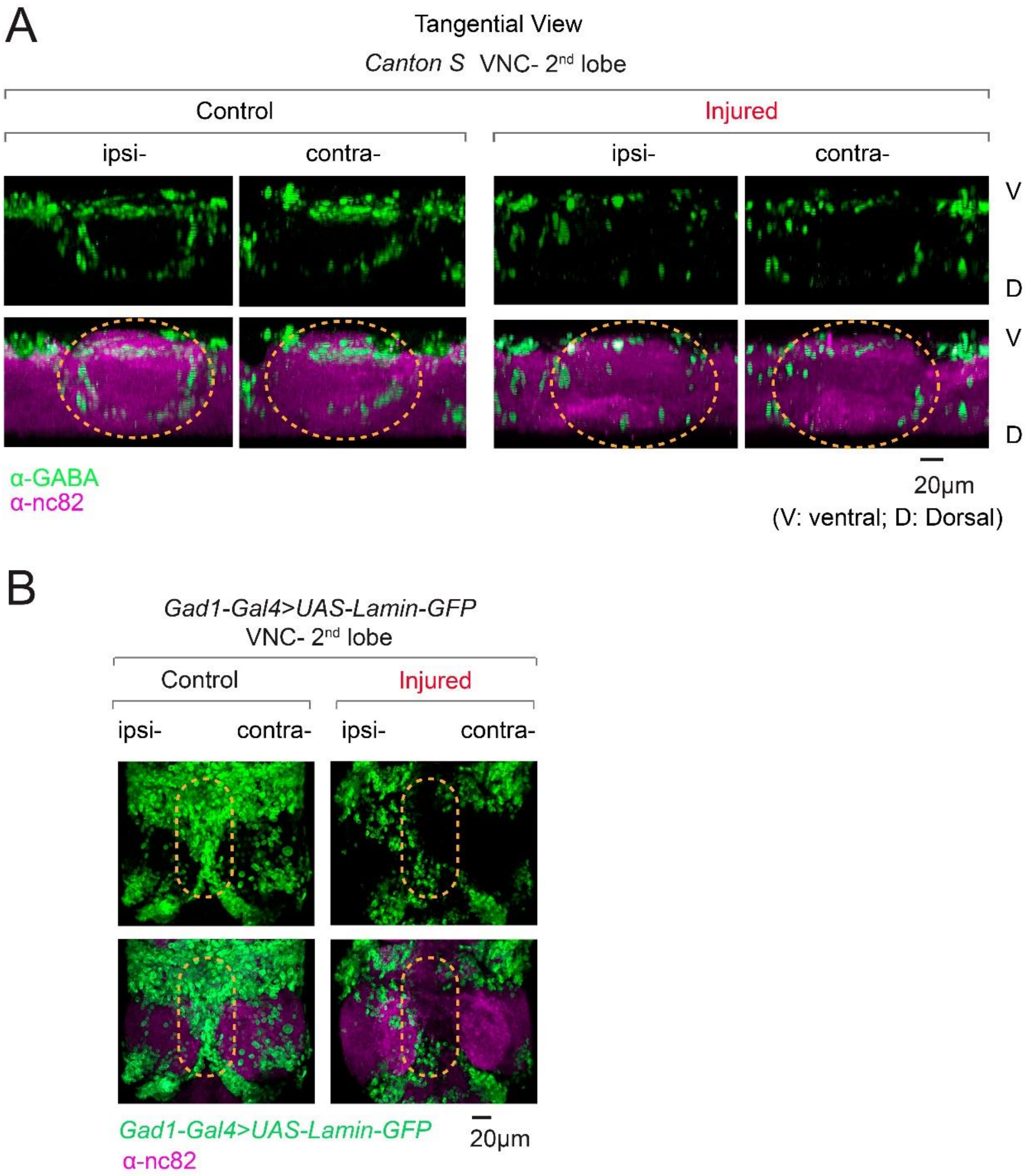
A
Tangential View
Canton S VNC- 2nd lobe
Control
Injured
ipsi-
contra-
ipsi-
contra-
V
D
V
D
α-GABA
α-nc82
20μm
(V: ventral; D: Dorsal)
B
Gad1-Gal4>UAS-Lamin-GFP
VNC- 2nd lobe
Control
Injured
ipsi-
contra-
ipsi-
contra-
Gad1-Gal4>UAS-Lamin-GFP
α-nc82
20μm

Fig. S4. Peripheral injury causes a loss of GABAergic interneurons. (A) Imaging of central GABAergic interneuron loss after peripheral injury; tangential view of VNC stained for GABA (green) and nc82 (magenta) from intact uninjured animals and injured animals (7 days after leg amputation), (n≥9). (B) Ventral top view of VNC 2nd lobe of flies expressing nuclei-labelled Lamin-GFP (green) in GABAergic interneurons (*Gad1-Gal4>UAS-Lamin-GFP*) and stained for nc82 (magenta) in intact uninjured animals and animals 7 days after peripheral injury(n≥9).

fig. S5

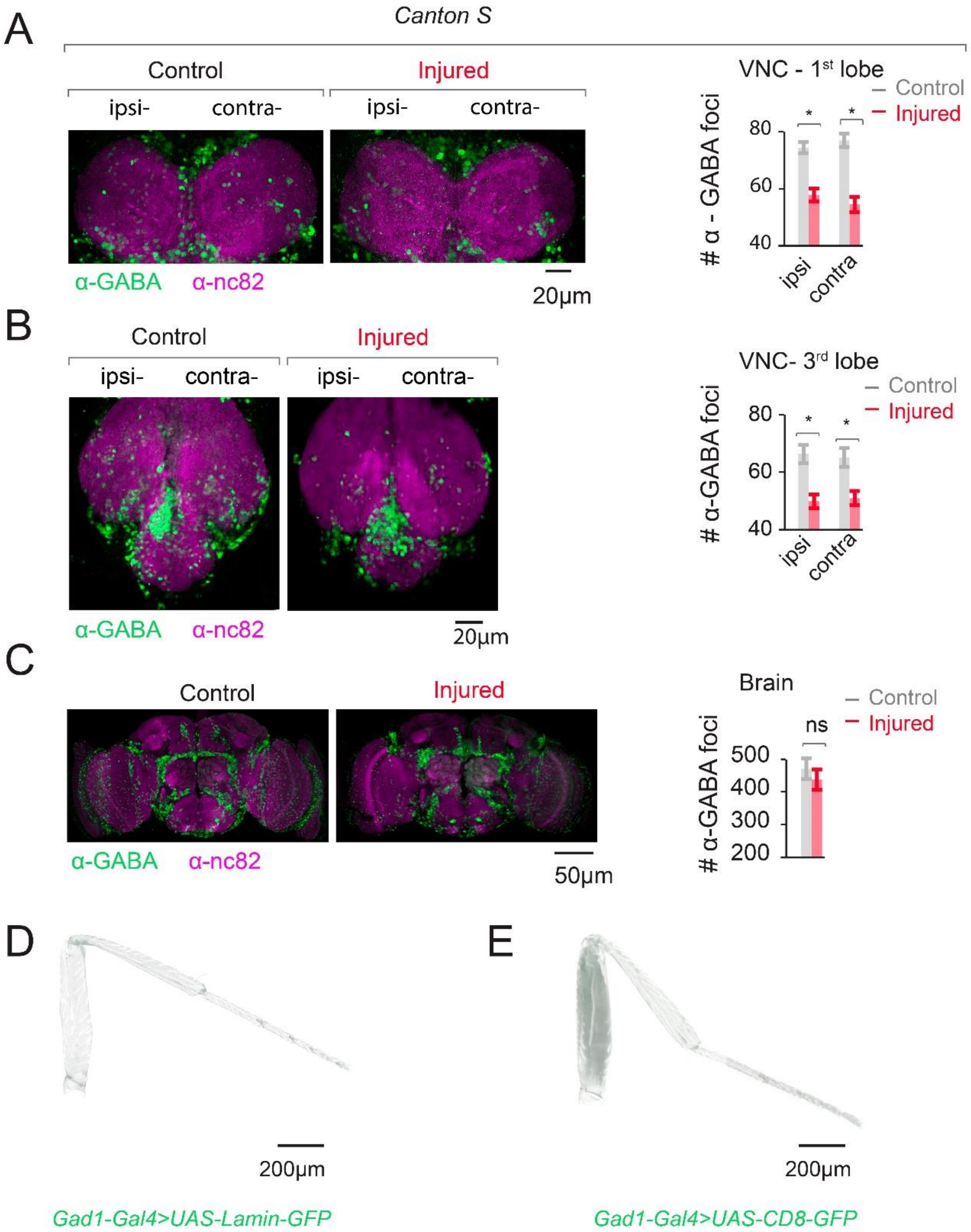

A
Canton S
Control
Injured
ipsi-
contra-
ipsi-
contra-
α-GABA
α-nc82
20μm
VNC - 1st lobe
Control
Injured
α - GABA foci
80
60
40
*
*
ipsi
contra
B
Control
Injured
ipsi-
contra-
ipsi-
contra-
α-GABA
α-nc82
20μm
VNC- 3rd lobe
Control
Injured
α-GABA foci
80
60
40
*
*
ipsi
contra
C
Control
Injured
α-GABA
α-nc82
50μm
Brain
Control
Injured
ns
α-GABA foci
500
400
300
200
D
200μm
Gad1-Gal4>UAS-Lamin-GFP
E
200μm
Gad1-Gal4>UAS-CD8-GFP

Fig. S5. Peripheral injury causes reduction in GABA in the VNC but not the brain. (A - C) Imaging and quantification of GABA foci from of (A) 1st VNC lobe, (B) 3rd VNC lobe, and (C) brains from uninjured and injured animals VNC stained with anti-GABA (green) and co-stained with anti-nc82 in magenta, n ≥9. (D - E) Neither cell bodies nor projections of GABAergic neurons were observed in the middle leg of flies with *Gad1-Gal4* driving (D) *UAS-lamin-GFP* or (E) *UAS-CD8-GFP*, (n≥7).

Data are represented as mean ± SEM. *$p<0.05$; ns, not significant, student's *t*-test.

fig. S6

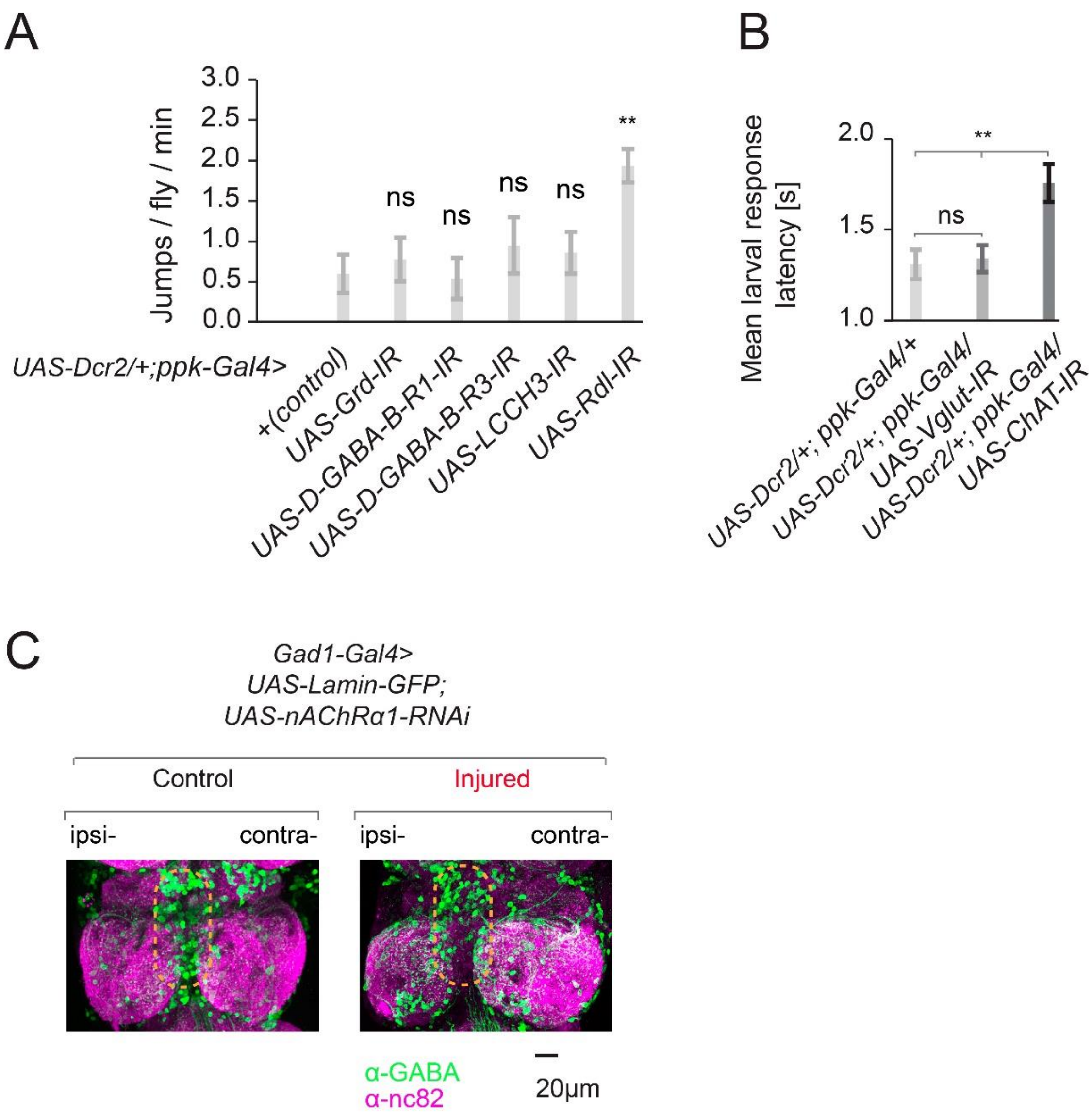
A
3.0
2.5
2.0
1.5
1.0
0.5
0.0
Jumps / fly / min
ns
ns
ns
ns
**
UAS-Dcr2/+;ppk-Gal4>
+(control)
UAS-Grd-IR
UAS-D-GABA-B-R1-IR
UAS-D-GABA-B-R3-IR
UAS-LCCH3-IR
UAS-Rdl-IR
B
2.0
1.5
1.0
Mean larval response latency [s]
**
ns
UAS-Dcr2/+; ppk-Gal4/+
UAS-Dcr2/+; ppk-Gal4/ UAS-Vglut-IR
UAS-Dcr2/+; ppk-Gal4/ UAS-ChAT-IR
C
Gad1-Gal4>
UAS-Lamin-GFP;
UAS-nAChRα1-RNAi
Control
Injured
ipsi-
contra-
ipsi-
contra-
α-GABA
α-nc82
20μm

Fig. S6. Knockdown of *Grd*, *GABA-B-R1*, or *GABA-B-R3* does not cause allodynia in uninjured flies, cholinergic output from *ppk*+ neurons mediates acute nociception behavior, and Twist is important for GABA loss after injury and mediates heat allodynia. (A) Nociceptive sensory neuron-specific (*ppk-Gal4*) knockdown of *GRD*, *GABA-B-R1*, or *GABA-B-R3* does not cause thermal allodynia (38°C) in uninjured flies (n≥9 replicates, 10 animals per replicate). (B) Larval heat response following RNAi knockdown of vesicular glutamate transport (*ppk-Gal4>UAS-Vglut* RNAi) or acetylcholine synthesis (*ppk-Gal4>UAS-ChAT* RNAi), (n≥60). (C) Knockdown of nAChRα1 in GABAergic neurons (*Gad1-Gal4> nAChRα1-IR*) prevents GABA loss after injury. Data are represented as mean ± SEM. ***p*<0.01; ns, not significant; two-way ANOVA followed by Tukey's post hoc test.

Supplementary Movies

Movie S1. Uninjured wild-type animals exhibit escape behavior in response to temperatures of ≥42°C. Flies are housed in each transparent test chamber and heated surface is set to 38°C (left), and 42°C (right). Uninjured intact control *Canton S* exhibit no jumps at 38°C, indicated by a continuous trajectory; but exhibit multiple jumps at 42°C, indicated by a discontinuous trajectory that shows colour changes when the fly jumps.

Movie S2. Peripheral injury causes increase in thermal allodynia in wild-type flies. When exposed to 38°C heated surface, uninjured intact control *Canton S* (left) exhibit no jumps, indicated by a continuous trajectory; while *Canton S* flies 7 days after injury (right) exhibit multiple jumps, indicated by a discontinuous trajectory that shows colour changes when the fly jumps.

Table S1. List of antibodies used in immunochemical experiments.

Antibodies	Source	Identifier	Dilution	Application
Anti-nc82	DSHB	CAT#nc82 RRID:AB_2314866	1:75	IF
Anti-GABA	Sigma	CAT#A2052 RRID:AB_477652	1:500	IF
Anti- cleaved caspase-3	Cell Signaling Technology	Cat# 9661 RRID:AB_2341188	1:500	IF
Anti-Twist1/2	GeneTex	CAT#GTX127310	1:500	IF
Goat anti-Rabbit IgG (H+L)-AF 488	ThermoFisher	CAT# A27034 RRID:AB_2536097	1:500	IF
Goat anti-Mouse IgG (H+L)-AF 555	ThermoFisher	CAT#A-21424 RRID:AB_141780	1:500	IF
Goat anti-Rabbit IgG (H+L) -AF647	ThermoFisher	CAT #A27040 RRID:AB_2536101	1:500	IF
Goat anti-Mouse IgG (H+L) -AF647	ThermoFisher	CAT#A-21236 RRID:AB_2535805	1:500	IF

Table S2. Detailed data of behavioral, immunochemical, and electrophysiological experiments.
